# Effect of temperature and its interaction with other meteorological factors on bacillary dysentery in Jilin Province, China

**DOI:** 10.1017/S0950268821000893

**Published:** 2021-04-22

**Authors:** Yingshuang Wang, Meina Li, Zhongqi Li, Ruiyu Chai, Xinxin Dong, Han Xu, Jin Wang, Laishun Yao, Yang Zhang, Qinglong Zhao, Yan Yao

**Affiliations:** 1 Department of Epidemiology and Biostatistics, School of Public Health, Jilin University, No. 1163 Xinmin Street, Changchun, Jilin 130021, China; 2 Department of Infection Control, The First Hospital of Jilin University, Changchun, Jilin 130021, China; 3 Jilin International Travel Healthcare Center, Changchun, Jilin 130062, China; 4 Emergency Office of Jilin Provincial Center for Disease Control and Prevention, Changchun, Jilin 130062, China

**Keywords:** Bacillary dysentery, interaction, meteorological factors, temperature

## Abstract

Bacterial dysentery (BD) brings a major disease burden to developing countries. Exploring the influence of temperature and its interaction with other meteorological factors on BD is significant for the prevention and early warning of BD in the context of climate change. Daily BD cases and meteorological data from 2008 to 2018 were collected in all nine prefecture-level cities in Jilin Province. A one-stage province-level model and a two-stage city-specific multivariate meta-pooled level distributed lag non-linear model were established to explore the correlation between temperature and BD, then the weather-stratified generalised additive model was used to test the interaction. During the study period, a total of 26 971 cases of BD were developed. The one-stage and two-stage cumulative dose-response ‘J’ curves overlapped, and results showed a positive correlation between temperature and BD with a 1–6 days lag effect. Age group ⩾5 years was found to be more sensitive to the effects. Moreover, there was a significant interaction between temperature, humidity and precipitation (*P* = 0.004, 0.002, respectively) on BD under high temperature (>0 °C), reminding residents and policymakers to pay attention to the prevention of BD in situations with both high temperature and humidity, high temperature and precipitation during the temperate monsoon climate.

## Introduction

Bacillary dysentery (BD) is an intestinal infectious disease caused by *Shigella*, which can be transmitted through contaminated water, food and human-to-human contact [1]. According to statistics, there were around 188 million cases of BD in 2010, 99% of which came from developing countries [2], and 164 300 people died of *Shigella* each year, of which children under 5 years of age were accounted for 33.41% [3], resulting in a great disease burden in low- and middle-income countries. China, as the largest developing country, although its incidence rate of BD has slowed down in recent years, still has a large number of BD cases. The onset of BD presents obvious seasonality, with a peak incidence in summer and autumn each year [1]. At the same time, the growth and reproduction of pathogenic bacteria, the eating habits and the immunity of human beings are both very easily affected by external climatic factors [4, 5]. Many studies have explored the relationship between climate factors and BD [6–11], and most of those chose temperature as the variable of interest. In addition to the high correlation between temperature and BD, the threat of global warming is also a contributing factor to the above phenomenon [7]. However, the effects of meteorological factors (MFs) on diseases often have complex interactions with each other [12, 13]. Under the global warming environment, identifying the impact of temperature and its modifiers on BD will have important practical significance for the prevention, control and early warning of this burdensome disease. Meanwhile, latitude is an important influencing factor of meteorology [14]. Some studies have suggested that the effects of MFs on diseases are not the same under different climate conditions [15], and the existing studies of BD are concentrated mostly in low-latitude cities in China, which leaves the data in high-latitude regions to be under reported.

Based on the above background, research on this subject is proposed. The aim is to explore the influence of temperature and its interaction with other MFs on BD in Jilin Province, a high-latitude region of China, and to determine the lag effects and sensitive populations. This present study may shed light upon providing important information mainly for the purpose of prevention, control and early warning of BD in the context of climate change.

## Methods

### Study location

**Fig. 1.**
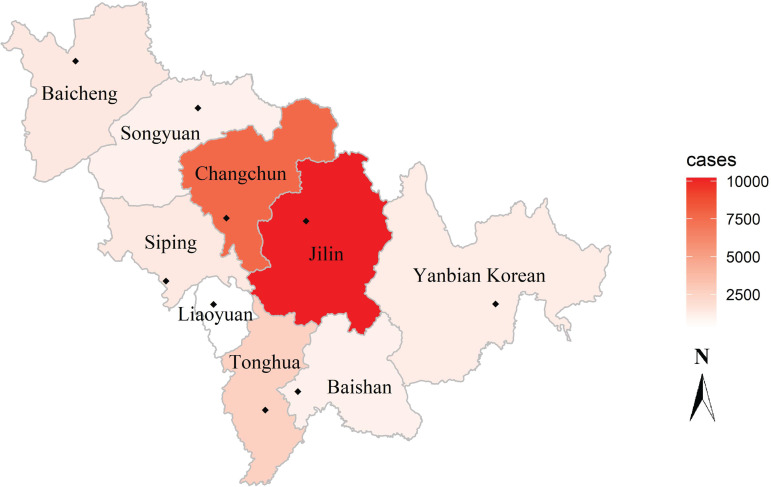
Map of Jilin province in China, panning between 121°38′ and 131°19′ east longitude and 40°50′ and 46°19′ north latitude. The depth of the colour represents the total number of cases of bacillary dysentery in 2008–2018, and the ‘◆’ represents the location of the weather station in each city.

Jilin Province is located in the geometric central zone of Northeast Asia, composed of Japan, Russia, North Korea, South Korea, Mongolia and northeastern China (spanning between 121°38′ and 131°19′ east longitude and 40°50′ and 46°19′ north latitude), has jurisdiction over eight prefecture-level cities (including Changchun, Jilin, Siping, Liaoyuan, Tonghua, Baishan, Songyuan and Baicheng) and one autonomous prefecture (Yanbian Korean Autonomous Prefecture) (Fig. 1). It covers an area of 187 400 square kilometres and has a total population of 26 907 300. Jilin Province has a temperate continental monsoon climate, with four distinct seasons every year, rain and heat over the same period.

### Data collection

The anonymous data of BD cases in Jilin Province from January 2008 to December 2018 came from the National Infectious Disease Reporting Information Management System collected by Jilin Provincial Center for Disease Control and Prevention. In China, BD is a class B infectious disease. Medical institutions at all levels are required to fill in the information online within 24 h after receiving the patients. The diagnostic criteria were based on Diagnostic Criteria for Bacterial and Amoebic Dysentery (WS 287-2008) [16]. The monitoring data mainly include the date of birth, date of onset, gender, residential address code etc.

The daily meteorological data covering the same period of each city in Jilin province came from the China Meteorological Data Sharing Service System (http://cdc.cma.gov.cn/), such as daily mean temperature, mean relative humidity, mean atmospheric pressure, mean wind speed, accumulated precipitation and sunshine hours. The location of representative meteorological stations in each city is shown in Figure 1. The nearest city of the same latitude meteorological data was used to fill in the missing data (the missing rate was 0.89%, of which 95% was missing in the accumulated precipitation) [17].

The demographic data of each city in the same period came from the statistical yearbook of Jilin Province (http://tjj.jl.gov.cn/tjsj/tjnj/).

### Statistical analysis

A distributed lag non-linear model (DLNM) was established to explore the potential non-linear lag relationship between temperature and BD [18, 19]. The weather-stratified generalised additive model (GAM) was used to explore the modification factors of temperature on BD.

#### Selecting MFs to be included in the model

The Spearman correlation matrix was established between the daily MFs and the incidence of BD, and the MFs statistically related to BD were selected to be included in the model. To avoid multicollinearity, one of the two MFs with high correlation (*r* > 0.7) was reasonably excluded.

#### Estimating the temperature–BD relationship at city and province levels

Firstly, a one-stage GAM combined with DLNM was established to explore the potential non-linear lag relationship between temperature and BD. At this time, we calculated the daily mean value of each meteorological index of nine prefecture-level cities weighted by annual average population to represent the meteorological distribution of the whole Jilin Province. Secondly, we used a two-stage DLNM to verify the accuracy of the above results. At this time, eight city-specific temperature–BD relationships were established (due to the small number of cases in Liaoyuan, it was combined with the neighbouring city Siping for modelling) at the first stage, then a multivariate meta-analysis was used to combine the eight city-specific effect values to gain a province-level effect at the second stage [20]. Cochran's *Q*-test and *I*^2^ statistics were applied to evaluate heterogeneity across cities. The model was established as follows:1
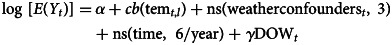
where *Y_t_* is the number of BDs on day *t* (a quasi-Poisson distribution was used to control overdispersion); *α* represents the intercept; a flexible cross-basis(*cb*) defined by the natural cubic spline function (ns, degree of freedom (df) = 3) for both space of temperature and lags was established to estimate the lag and complex non-linear effect of daily mean temperature on BD incidence. According to the rapid onset of BD and the short-term impact of MFs on the pathogen, the maximum number of lag days was set up to 7 days [5, 21]. The ns (df = 3) was used to adjust the potential non-linear confounding effect of other MFs (including *relative humidity, wind speed, precipitation, sunshine hours*). *Time* is a newly set time ordinal variable, which was introduced into ns to control seasonality and long-term trend, and based on generalised cross-validation (GCV) criterion, the df was 6 per year; DOW*_t_* is a dummy variable to control the day of the week effect.

After the temperature-lag–BD effect model was established, 0 °C was used as the reference temperature to evaluate the influence of different temperatures on BD and lag effect. The effect size was expressed by the relative risk (RR). According to the stratification of gender and age to explore the temperature-sensitive population, and the logRR *z*-test method proposed by Altman and Bland [22] was used to compare the differences in overlapping confidence intervals between stratified groups.

#### Estimating the interaction between temperature and other MFs

A stratification GAM was applied to examine the interaction effect between temperature and other MFs on BD. The moving average of temperature in effective lag days was incorporated into the model as a continuous variable, whereas other corresponding meteorological variables were transformed into binary variables by taking the average value as the dividing point. When the interaction between a certain meteorological factor and temperature was being examined, the remaining meteorological variables were incorporated into the model as continuous covariates. The model was established as follows:2
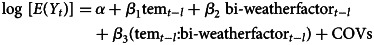
where tem*_t−l_* represents the moving average of *l*-day temperature with significance lag effect; bi-weatherfactor*_t−l_* represents the binary MFs interacting with temperature; *β*_1_–*β*_3_ represent the respective regression coefficients, among which, if *β*_3_ was statistically significant indicating the existence of interaction; COVs are the covariates of adjustment. See model (1) for details. The df was selected according to the minimum principle of GSV. The DLNM results suggested that the logRR of BD and temperature were approximately linear with different slopes on both sides of 0 °C, therefore, the effect value of the meteorological stratification model was expressed as the RR of BD incidence for per 5 °C increase in temperature above and below 0 °C.

Sensitivity analyses of main findings were conducted by (1) changing the df of ns for weather variables (3, 4) and time (5–8 per/year), (2) changing the maximum lag days (5 d, 7 d, 10 d, 14 d) and (3) testing residual distribution. All analyses were conducted in R (version 3.6.3). All statistical tests were two-sided with a 0.05 significance level.

## Results

### Descriptive analysis

**Fig. 2.**
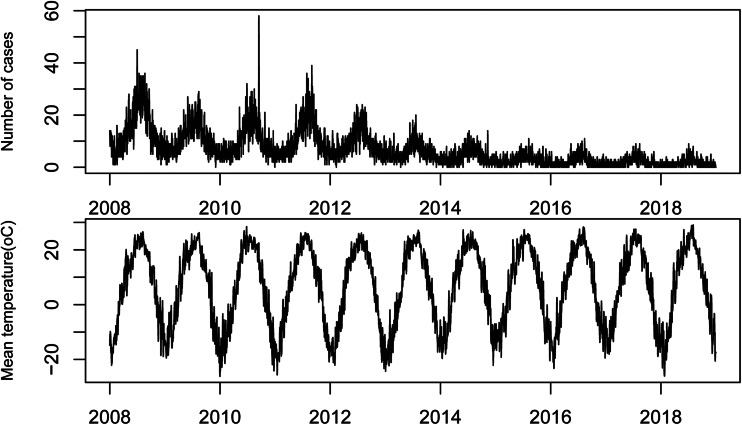
Time series plot of daily temperature and bacillary dysentery in Jilin province, China, 2008–2018.

From 1 January 2008 to 31 December 2018, there were 26 971 BD cases in Jilin Province, including 15 686 males (58.2%), 11 285 females (41.8%), and 46.12% patients younger than 5 years old. A total number of cases from high to low were Jilin (10 213), Changchun (7716), Tonghua (2645), Baicheng (1454), Siping (1427), Yanbian Korean (1241), Songyuan (1029), Baishan (1028) and Liaoyuan (218) (Fig. 1). The daily BD cases and meteorological distribution are shown in Table 1. The peak incidence of BD was from June to July each year. Since 2012, the number of BD cases has decreased year by year (Fig. 2).

**Table 1.** Distribution of daily bacillary dysentery cases and meteorological factors in Jilin Province, China, 2008–2018

Variables	Mean	Min	P25	P50	P75	Max
Daily bacillary dysentery cases						
Total	6.7	0	2	5	9	58
Male	3.9	0	1	3	6	29
Female	2.8	0	1	2	4	29
>5 years old	3.6	0	1	2	5	48
⩽5 years old	3.1	0	1	2	4	19
Daily mean temperature (°C)	6.2	−26.2	−6.9	8.6	19.2	29.1
Daily mean relative humidity (%)	62.7	19.3	53.2	64.0	73.5	96.0
Daily mean pressure (kPa)	98.7	95.8	98.1	98.7	99.3	101.2
Daily mean wind speed (m/s)	2.5	0.59	1.8	2.4	3.0	7.4
Daily precipitation (mm)	1.9	0	0	0.1	1.5	61.7
Daily sunshine hours (h)	6.5	0	4.2	7.0	9.0	13.2

The provincial daily meteorological value is the mean value weighted by the population of each city.

### Temperature-lag–BD incidence effect

**Fig. 3.**
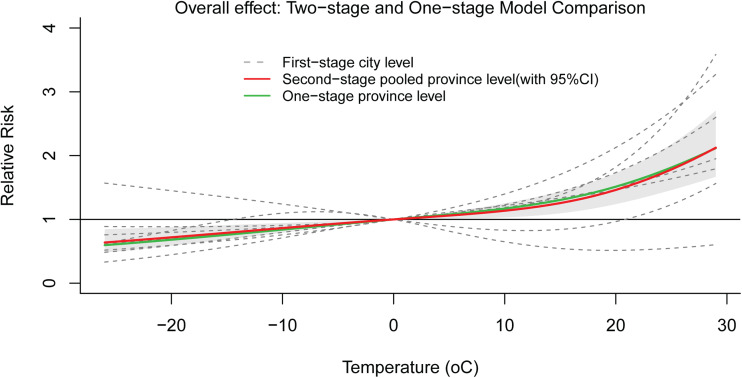
Overall cumulative effects between daily mean temperature and BD incidence.

**Fig. 4.**
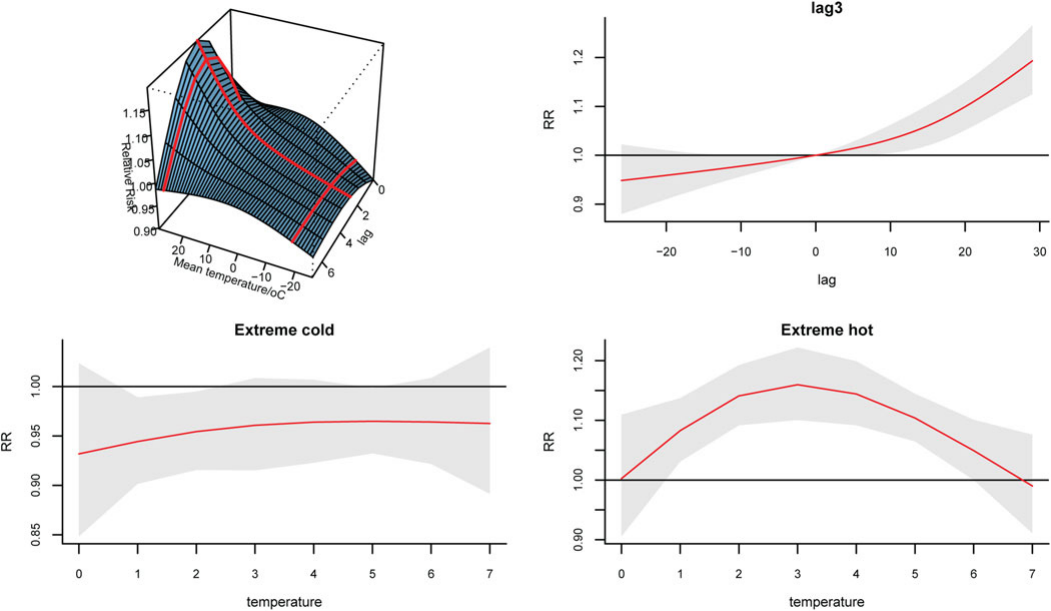
Temperature-lag–BD incidence relative risk 3D diagram and its slices.

**Fig. 5.**
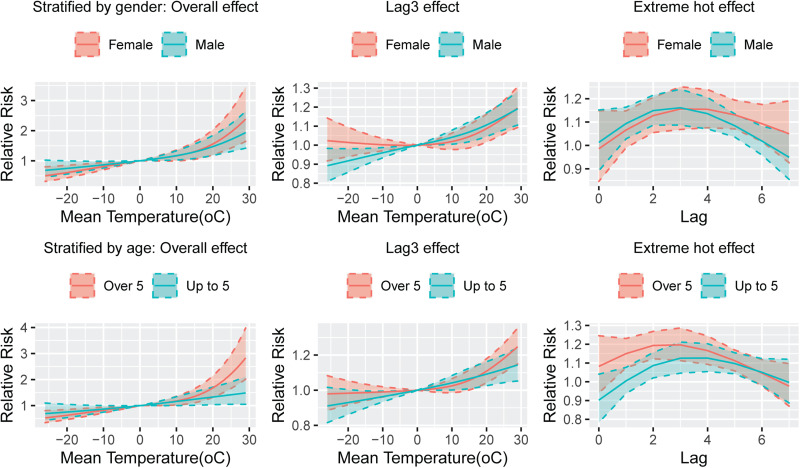
The plot of temperature–BD relationship stratified by gender and age under different conditions.

The Spearman correlation matrix showed that temperature, relative humidity, precipitation, wind speed and sunshine hours were all statistically related to the number of daily BD incidences, however, there was a strong negative correlation between atmospheric pressure and temperature (*r* = −0.76, Fig. S1). Thus, the atmospheric pressure was excluded and the remaining MFs were included in the model. The fitting curve of the cumulative effect of temperature on BD lagging for 7 days was nearly completely overlapped at one-stage and two-stage province levels (Fig. 3). Moreover, the heterogeneity among prefecture-level cities was very small (*Q* = 19.45, *I*^2^ = 1.0%, *P* = 0.556), indicating that the results were relatively stable. The relationship between temperature and the RR of BD showed a positive correlation with a ‘J’ curve. We defined the daily average temperature of P97.5 (26 °C) as extremely hot, and P2.5 (−19 °C) as extreme cold. Compared with 0 °C, the overall cumulative RRs of extreme hot and extreme cold were 1.88 (95% CI 1.51–2.34) and 0.70 (95% CI 0.56–0.86), respectively, after adjusting for other MFs and seasonality and long-term trends. The temperature-lag–BD incidence 3D diagram and its slices indicated that as the temperature increases, the RR value continues to increase. There was a lag effect in the influence of extreme hot on BD, which manifested as the effect started at lag 1 day, reached a maximum at lag 3 days, and continued until lag 6 days. The extreme cold temperature had a protective effect on the lag time of 1–2 days (Fig. 4). See Figure 5 for the results of stratification by gender and age. To compare whether the overlapping confidence intervals were significantly different, the pairs of RRs with the largest difference in the figure were extracted for the log transformation *t*-test. The results indicated that the extreme hot temperature effect under a lag 1-day condition and the overall cumulative effect of the age group over 5 years old were both greater than under 5 years old (*P* = 0.02, 0.006 respectively). There was no significant difference between the various conditions of gender stratification (Table 2).

**Table 2.** The relationship between temperature and bacillary dysentery stratified by gender and age under different conditions

Group	RR (95% CI)^a^	*z*	*P*
Overall effect: P97.5 (26 °C)			
Female	2.05 (1.48–2.85)		
Male	1.76 (1.34–2.32)	0.696	0.5
Over 5	2.36 (1.74–3.18)		
Up to 5	1.43 (1.05–1.95)	2.249	**0**.**02**
Lag 3 effect: P2.5 (−19 °C)			
Female	1.01 (0.94–1.09)		
Male	0.92 (0.87–0.98)	1.95	0.052
Extreme hot effect: Lag 7			
Female	1.05 (0.93–1.19)		
Male	0.95 (0.85–1.05)	1.204	0.23
Extreme hot effect: Lag 1			
Over 5	1.15 (1.07–1.23)		
Up to 5	1.00 (0.94–1.08)	2.735	**0**.**006**

a 0 °C was the reference temperature. Adjusted wind speed, relative humidity, accumulated precipitation, sunshine hours, seasonality and long-term trends and day of week effects.

### Interaction between temperature and other MFs on BD

**Fig. 6.**
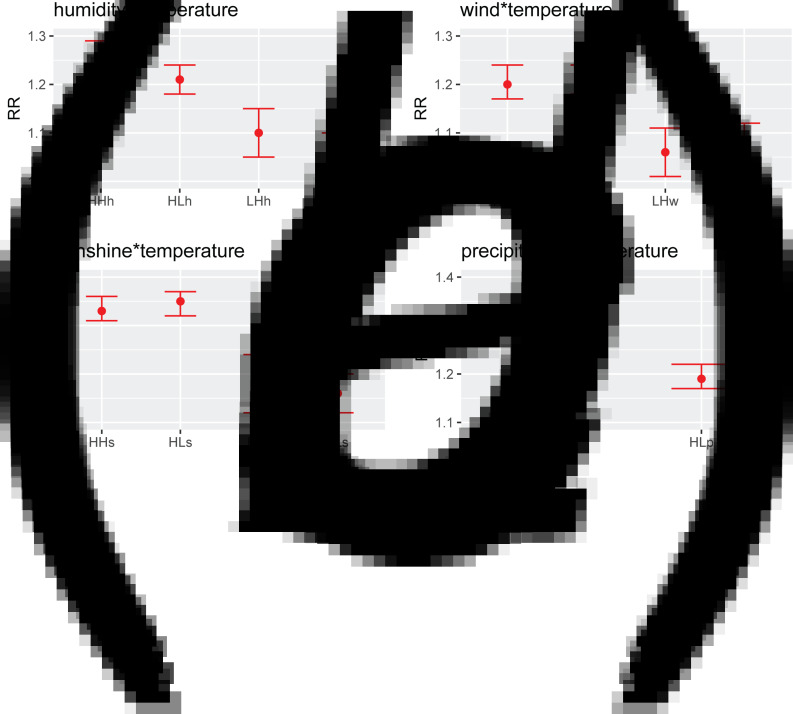
The relative risk of bacillary dysentery for every 5 °C increase in temperature under different climatic conditions. There was almost no rainfall under low-temperature conditions, and it was impossible to carry out a stratified analysis of precipitation. HHh, high temperature and high humidity; HLh, high temperature and low humidity; LHh, low temperature and high humidity; LLh, low temperature and low humidity; HHw, high temperature and high wind speed; HLw, high temperature and low wind speed; LHw, low temperature and high wind speed; LLw, low temperature and low wind speed; HHs, high temperature and high sunshine; HLs, high temperature and low sunshine; LHs, low temperature and high sunshine; LLs, low temperature and low sunshine; HHp, high temperature and high precipitation; HLp, high temperature and low precipitation.

The weather stratification GAM results suggested that under high temperature (>0 °C), there was a significant interaction between temperature, humidity and precipitation (*P* = 0.004, 0.002, respectively) on BD, but the absence of interaction with wind speed and sunshine hours (*P* = 0.497, 0.898, respectively). Under low temperature (<0 °C), the effect of temperature was not modified by other MFs (*P* = 0.819, 0.790, 0.711, 0.654, respectively). When the weather was characterised by high temperature and high precipitation, the temperature made the greatest impact on BD. For every 5 °C increase in temperature, the RR of BD was 1.27 (95% CI 1.24–1.31). Followed by high temperature and high humidity conditions, temperature per 5 °C increase, the RR of BD was 1.26 (95% CI 1.23–1.29) (Fig. 6).

### Sensitivity analysis

After changing the df of the model and the maximum lag days, the main effect between temperature and BD did not change, and residuals showed a normal distribution, which proved the robustness of the results (Figs S2–S5).

## Discussion

This study used the 2008–2018 BD incidence data in Jilin Province from the China Infectious Disease Surveillance System and the meteorological daily data during the same period, and established GAM and DLNM to explore the effect of temperature and its interaction with other MFs on BD. The results revealed that temperature had a positive correlation with the incidence of BD. Humidity and precipitation were modifiers between temperature and BD. High temperature combined with high humidity or high precipitation weather conditions were conducive to the onset of BD.

Our study suggested that the temperature was positively correlated with the risk of BD. It is consistent with the research results based on Korea [8], Vietnam [23], Beijing [10], Lanzhou [24], Jinan [21], Wuhan [25], Anhui [17], Changsha [26] and Guangzhou [27]. A warm and suitable water environment is conducive to the survival of pathogenic bacteria. At the same time, zooplankton and algae multiply rapidly, creating conditions for the unlimited growth and reproduction of bacteria [28]. Meanwhile, the high temperature changes of the weather will also directly affect the various systems of the body, reduce the function of the immune system, and increase the susceptibility to infection [26, 29]. Also, hot weather will affect the storage and processing of food, and change the frequency of people going out and eating habits. For example, due to the hot weather, drinking a lot of water will dilute stomach acid, which indirectly affects the susceptibility of humans to BD [25]. In Beijing, when the temperature was greater than 12.5 °C, there was a linear correlation between the temperature and the incidence of BD, and the excess risk (ER) for 1 °C increase was 1.06% (95% CI 0.63–1.49) [10]. In Wuhan, for a 1 °C increase in mean temperature, ER was 0.94% (95% CI 0.46–1.43) [25]. In the age group older than 65 in Korea, the incidence of shigellosis was expected to increase by 13.6% [8]. And each 1 °C rise of temperature corresponded to an increase of 3.60% (95% CI 3.03–4.18) in the monthly number of BD cases in Guangzhou [27]. In summary, the temperature and the incidence of BD were positively correlated to varying degrees in different countries and regions.

This study showed that the effect of temperature on BD appeared after a lag of 1 day, reached the maximum after 3 days, and maintained until the sixth day. It is consistent with the results of Li *et al*. [10, 25]. The average incubation period of BD is 1–2 days [30], coupled with the propagation time in the external environment which the results of our study are in line with biological rationality. Furthermore, our study also indicated that the effect of temperature on BD was not different between men and women, which is consistent with most current research to date. However, there were differences in temperature sensitivity among different age groups. The age group over 5 years old was more sensitive to temperature, which is consistent with the research results based on Anhui Province [17], but Wang *et al*. [24] found that the group less than 3 years old was a sensitive population, and Liu *et al*. [11] based on the whole China data, proved that age was not a modification factor of temperature on BD effect. The reason for the above discrepancy may be that the causative species/serotypes of shigellosis are not evenly distributed everywhere, and different species/serotypes are not the same sensitive to people of different ages [15, 31, 32].

A novel finding of this study is the significant interaction between humidity/precipitation and temperature, on BD incidence, that meant under high temperature and high humidity, high temperature and high precipitation weather conditions, the number of BD cases was higher, which is consistent with the research conclusions of Liu *et al*. [33]. They used principal component analysis and classification and regression trees found that when minimum temperature was at a high level, the high incidence of dysentery occurred if relative humidity or precipitation was at a high level. In order to clarify the combined effect of temperature and humidity on BD, Zhang *et al*. [34] constructed a Humidex, and the results pointed out that Humidex was positively correlated with the incidence of BD. High humidity can increase the survival time of *Shigella* on the food surface [35]. An etiological study also suggested that the function efficiency of reticuloendothelium decreased in high temperature and humidity environment, which in return increased the sensitivity to *Shigella* endotoxin [21]. The main route of transmission of BD is faecal-oral transmission, and water is an essential link in spreading the disease. Therefore, it is not surprising that there is an interaction between high temperature and precipitation. A large number of studies have shown that precipitation was positively correlated with BD incidence [10, 36, 37]. The environment of high temperature combined with high rainfall is not only conducive to the growth and reproduction of BD, but also conducive to the spread and transmission of BD.

Latitude is one of the important factors that determine the meteorological differences in various places [14]. Studies have shown that as the latitude decreases, the peak months of BD incidence move back, and the most noticed peak months of BD in North China were from June to September, and in South China, except for a few low-latitude cities, were from July to October [38, 39]. The location of this study is Jilin Province, spanning between 121°38′ and 131°19′ east longitude and 40°50′ and 46°19′ north latitude, a high latitude region in Northeast China with temperate monsoon climate, and the peak incidence of BD was in June and July, which is in line with the above rule. The results showed that there was no threshold effect of temperature on the incidence of BD in Jilin Province. A study by Wang *et al*. [40] revealed that the effective temperature thresholds for BD of Shenyang (41°44′N, 123°31′E, temperate monsoon climate), Beijing (39°48′N, 116°28′E, temperate monsoon climate), Chongqing (29°35′N, 106°28′E, humid subtropical monsoon climate) and Shenzhen (22°32′N, 114°E, subtropical to tropical transitional marine climate) were −3, 4, 9 and 16 °C, respectively, and the effect of temperature on BD gradually decreased in the corresponding city. Liu *et al*. [11] used data from 316 cities in China to calculate attributable risks for BD due to average daily mean temperatures above the 50th percentile, and we can see from the results the attribution risk in the south was higher than that in the northern part. It is suggested that as the latitude increases, the effective threshold of temperature decreases or even disappears, and the sensitivity of BD to temperature increases. To sum up, in different latitudes or climatic zones, the peak month of BD, the temperature threshold of BD and the effect of temperature on BD are different. Although the internal complex mechanism is still unclear, this also suggests the importance of making BD prevention and control strategies according to local conditions.

This study has the following strengths: Firstly, we used provincial daily data to construct a DLNM and pooled eight city-level effect values for verification, which increased the daily incidence sample size and made the results more reliable. Secondly, few articles have examined the impact of the interaction effect between MFs in high latitudes in China on BD. Our research adds to this field. The research has the following limitations: Firstly, because most patients with BD are mild cases, some people do not seek medical treatment and heal naturally by themselves, which leads to underreporting and potential selection bias. Secondly, some possible confounding factors that not only affect the incidence of BD, but also related to MFs are not included in the model for adjustment, such as people's lifestyle, eating habits etc., leading to a potential confounding bias.

There was an obvious positive correlation between temperature and BD incidence with a lag effect in Jilin Province, China. The age group older than 5 years was more sensitive to the effects of temperature. Humidity and precipitation could modify the effect of temperature on BD, which called attention to the health department to take preventive measures for BD under the conditions of high temperature and high humidity, high temperature and high precipitation in the temperate monsoon climate.

## Data Availability

The datasets analysed during the current study are available from the corresponding author on a reasonable request.

## Supplementary material

For supplementary material accompanying this paper visit http://dx.doi.org/10.1017/S0950268821000893. click here to view supplementary material
