# Supplementary material for: Effect of temperature and its interaction with other meteorological factors on bacillary dysentery in Jilin Province, China
Source: Epidemiol Infect. 2021 Apr 22;149:e121. doi: 10.1017/S0950268821000893 (PMC8161304; doi:10.1017/S0950268821000893)
Supplement: Supplementary file 1 [file S0950268821000893sup001.docx]

***Epidemiology and Infection***

**Effect of temperature and its interaction with other meteorological factors on bacillary dysentery in Jilin Province, China**

Yingshuang Wang, Meina Li, Zhongqi Li, Ruiyu Chai, Xinxin Dong, Han Xu, Jin Wang, Laishun Yao, Yang Zhang, Qinglong Zhao, Yan Yao

**Supplemental Materials**

**Figure S1** Spearman correlation matrix between the daily meteorological factors and the number of cases of bacillary dysentery

**Figure S2** Sensitivity analyses for one-stage model by changing the df of ns for weather variables

**Figure S3** Sensitivity analyses for one-stage model by changing the df/year of ns for time

**Figure S4** Sensitivity analyses for one-stage model by changing the maximum lag days

**Figure S5** Residual distribution and normal QQ plot


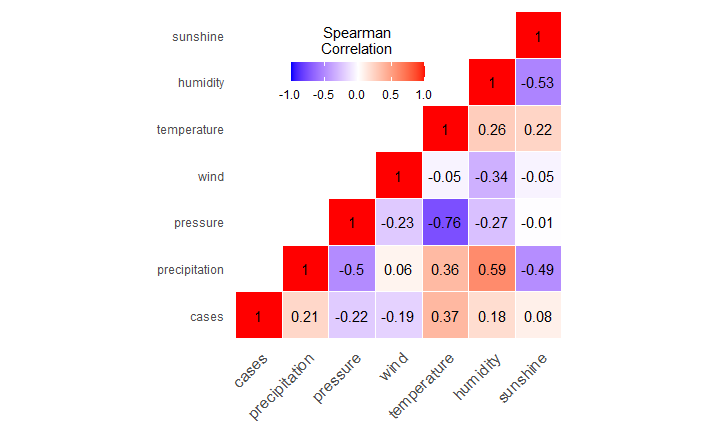


**Figure S1** Spearman correlation matrix between the daily meteorological factors and the number of cases of bacillary dysentery


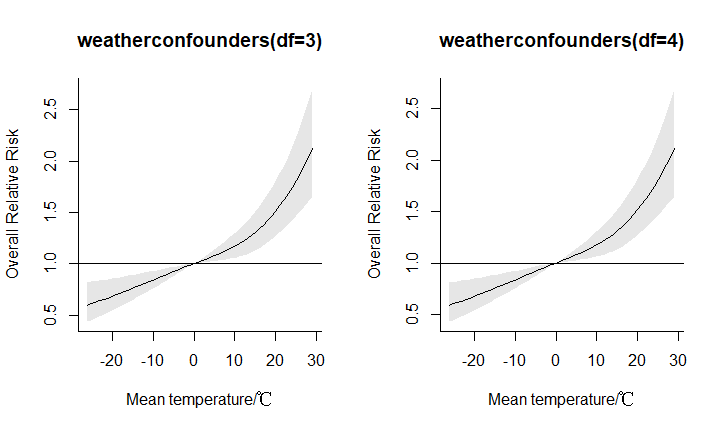


**Figure S2** Sensitivity analyses for one-stage model by changing the df of ns for weather variables


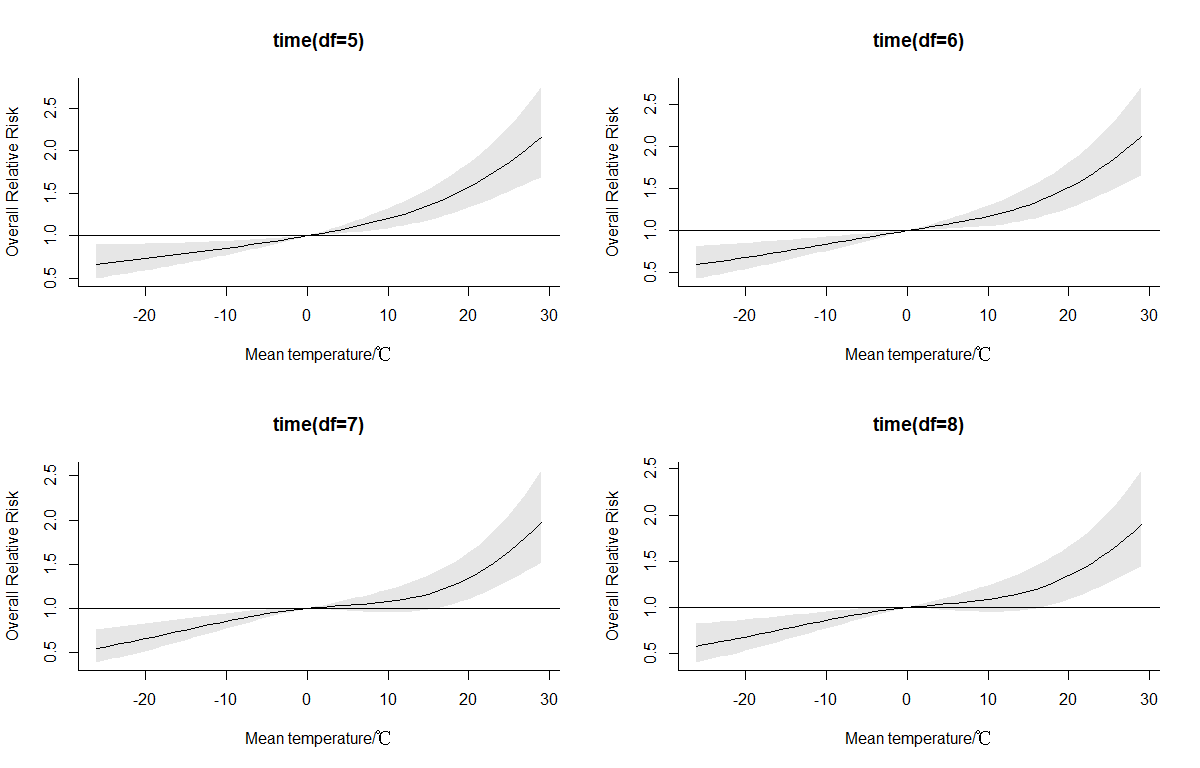


**Figure S3** Sensitivity analyses for one-stage model by changing the df/year of ns for time


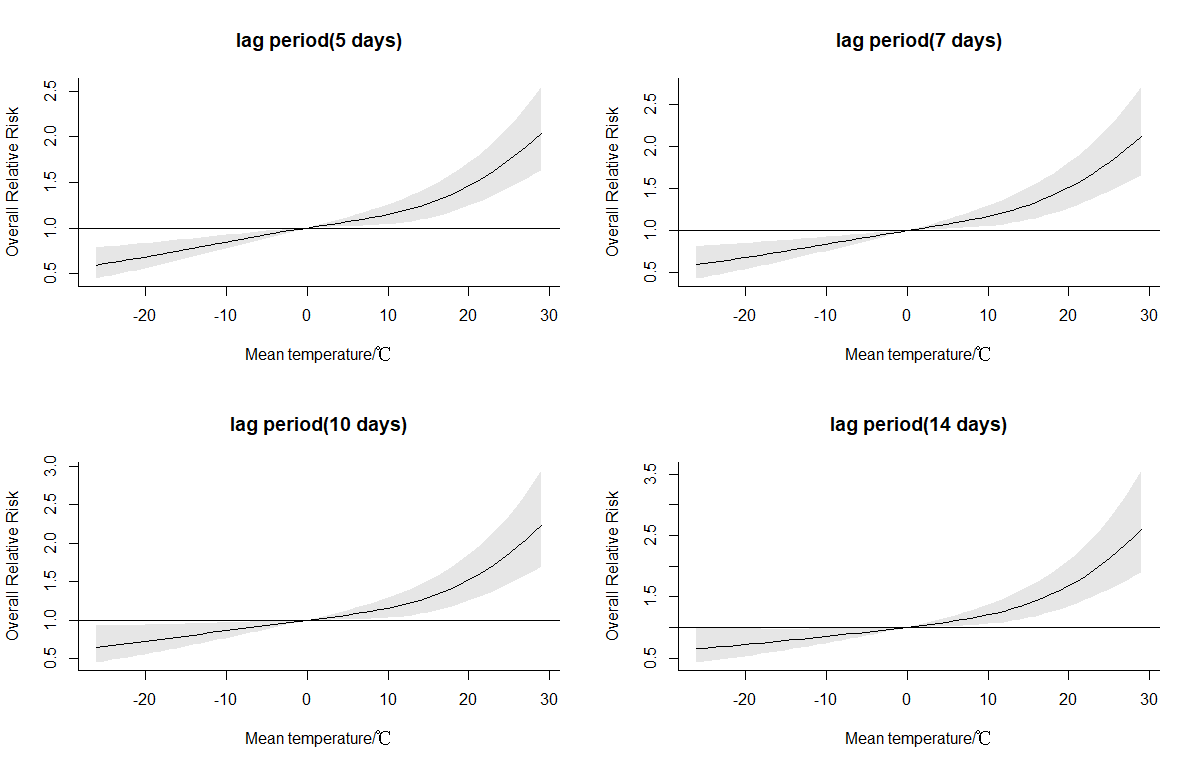


**Figure S4** Sensitivity analyses for one-stage model by changing the maximum lag days


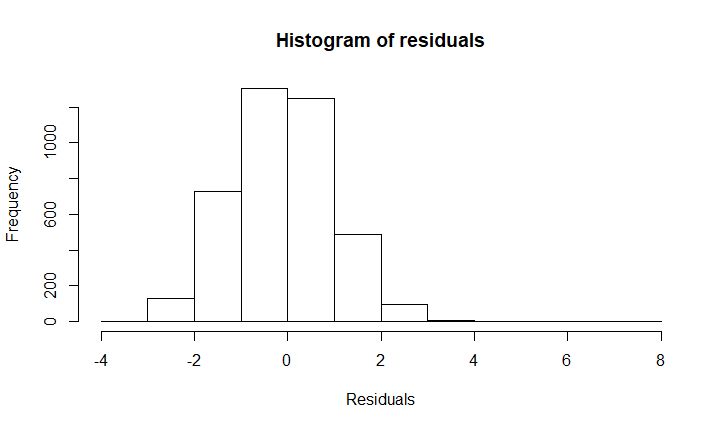

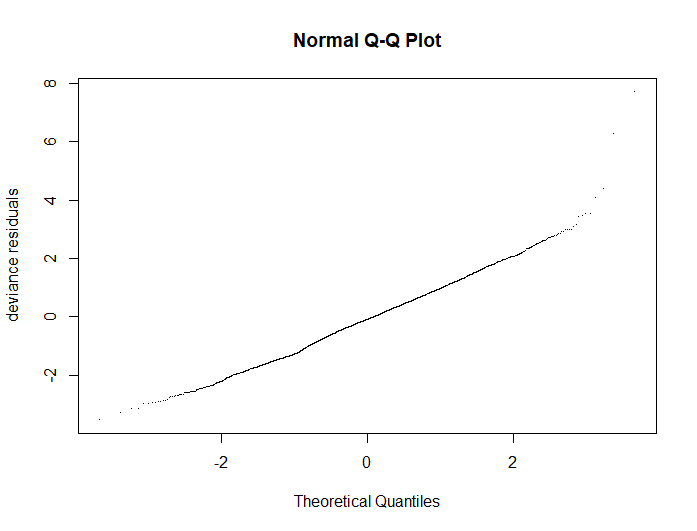


**Figure S5** Residual distribution and normal QQ plot
